# Chronic Diseases

**Published:** 1888-07

**Authors:** 


### HOMŒOPATHIC PHYSICIAN,

A MONTHLY JOURNAL OF

HOMŒOPATHIC MATERIA MEDICA AND CLINICAL MEDICINE.

"If our school ever gives up the strict inductive method of Hahnemann, we are lost, and deserve only to be mentioned as a caricature in the history of medicine."—CONSTANTINE HERING.

Vol. VIII. JULY, 1888. No. 7.

### CHRONIC DISEASES.

In recent issues of this journal brief papers have been published upon sycosis, syphilis, and psora as chronic miasms, and, hence, as factors in producing chronic diseases. The object of this present paper is to continue to attract interest to this important subject. We do not expect to produce any new ideas or startling theories on these diseases, but merely desire to cause them to be discussed and studied, believing that study of them cannot fail to benefit the practitioner.

Every observant physician has been puzzled at times to understand why it is that some cases of any given disease should be more difficult to cure than others. Usually this difficulty has been ascribed to some inherited dyscrasia or to idiosyncrasy and like indefinite terms. It remained for Hahnemann to give definite and satisfactory reasons for these stubborn cases. As we all know, Hahnemann was an inquisitive person ; he wanted to understand the true reasons for everything he observed. Seeing that Cinchona cured many cases of intermittent fever, he asked himself, Why is it? And the answer was the discovery of the Law of the Similars ; so, too, after observing that this law enabled him to cure speedily and thoroughly most cases of disease, Hahnemann again questioned himself, and asked, Why do not these remedies cure all disease alike promptly and permanently? The answer to this question was his grand discovery of the nature of these chronic diseases. This doctrine of chronic diseases cannot any longer be considered a mere theory; it is a fact based upon the multifold experience of many observers.

In the treatment of chronic diseases, as in all others, the symptoms, not the name, are to point out the proper remedy. Hahnemann teaches us both in the *Organon* and in his work on *Chronic Diseases* that the symptoms of the patient are to be the sole guide for the selection of the remedy, whether this remedy is to be an anti-psoric or an anti-syphilitic or anti-sycotic.

Hahnemann does not consider such diseases as are caused by occupation or residence as true chronic diseases, for these can be relieved by a change of residence or occupation. In his *Organon* he tells us, "The true natural chronic diseases are those which are produced by a chronic miasm, making continual progress in the body when no specific curative remedy is opposed to them, and which, notwithstanding all imaginable care, both with regard to the regimen of the mind and body, never cease to torment the patient with an accumulation of miseries that endure to the end of his existence." "Hitherto, syphilis was in some measure known as one of these chronic miasmatic diseases, which, being uncured, continued to the end of life. Sycosis, which likewise cannot be subdued by the vital powers alone, has never been regarded as a distinct species of chronic disease depending on an internal miasm, and it was supposed to be cured when the excrescences on the skin were destroyed, while no attention was paid to their internal source, which still continued to exist." Hahnemann further tells us that psora is the parent of all chronic diseases not caused by either syphilis or sycosis. According to his experience the effects of this miasm are practically unlimited. In the *Organon* he says: "But a chronic miasm that is incomparably greater and far more important than either of the two named, is *psora*. The other two disclose the specific internal affection whence they emanate—the one by chancres, the other by cauliflower excrescences. But it is not until the whole organism is infected that psora declares its huge internal chronic miasm by a cutaneous eruption (sometimes consisting of only a few pimples) that is wholly peculiar to it, accompanied by insupportable tickling, voluptuous itching, and a peculiar odor. This psora is the sole true and fundamental cause that produces all the other countless forms of disease under the name of nervous debility, hysteria, hemicrania, idiocy, hypochondriasis, insanity, melancholy, madness, epilepsy, spasms of all kinds, softening of the bones, caries, cancers, fungus-hæmatodes, gravel, gout, hæmorrhoids, dropsy, jaundice, ulcers, asthma, phthisis, cataract, deafness, menstrual irregularities, etc., all of which now appear in pathology as separate, distinct, and independent diseases." Such is Hahnemann's declaration; but educated, as most of us have been in the pathological teachings of the dominant school of medicine, to us these statements may seem exaggerated, yet to those of the homœopathic school who have had the greatest experience in treating chronic diseases, Hahnemann's words have been proven true. Those of our school who have in the past been most successful in curing those chronic diseases which are generally considered as uncurable have given the greatest time and attention to the study of Hahnemann's three chronic miasms, and it is evident that we must do likewise if we would emulate their success. Bœnninghausen, who was probably the most successful prescriber in chronic ailments our school has produced, was also the closest student of these chronic miasms. It is said that Bœnninghausen had a record of some three or four hundred cases of epilepsy which he had cured, a record which has certainly never been surpassed. Constantine Hering cured many chronic cases which other physicians could not help in the least; so, too, did Dr. Lippe. These men had no secret methods of practice, no secret remedies; they had nothing more than the humblest of us may acquire, a profound knowledge of Hahnemann's *Organon*, of his *Chronic Diseases*, and of the homœopathic *Materia Medica*. Cannot we all obtain that knowledge also, and having it, cannot we, too, use it to cure these scourges of the human race as they did? The treatment of acute diseases is comparatively simple, the selection of the remedy is generally easy, and the result follows so promptly that one cannot be long in doubt as to the result of his prescription. But in chronic cases the selection of the remedy is very difficult (the past, as well as the present, history of the case must be studied), and the action following its administration is necessarily slow and tedious. Hence, it requires wisdom to select the remedy for chronic cases, and great patience, combined with acute observation, to watch the case in order to observe the curative process.

In chronic cases one might select the proper remedy every time with unerring accuracy, yet he would most assuredly fail to cure a single (chronic) case if he did not know how to give his remedy. Too frequent repetition or too sudden change of remedies would as certainly prevent a cure as would the giving of the wrong drug. To cure these diseases one must be thoroughly well versed in homœopathic philosophy ; an accurate and complete knowledge of the materia medica alone is not sufficient for treating chronic diseases. In order to help us to judge of the action of our remedies in such cases as these, I will quote some words of advice written by Hering. In his preface to Hahnemann's first volume on *Chronic Diseases*, Hering said :

" As acute diseases *terminate* in an eruption on the skin, which divides, dries up, and then passes off, so it is with many chronic diseases. All diseases diminish in intensity, improve, and are cured by the internal organism freeing itself from them little by little. The internal disease approaches more and more to the external tissues until it finally arrives at the skin.

" Every homœopathic physician must have observed that the improvement in pain takes place from above—downward—and in diseases from within—outward. This is the reason why chronic diseases, if they are thoroughly cured, always terminate in some cutaneous eruption, which differs according to the different constitutions of the patients. This cutaneous eruption may even be perceived when a cure is impossible, and even when remedies have been improperly chosen. The skin being the outermost surface of the body, it receives upon itself the extreme termination of the disease. This cutaneous eruption is not a morbid secretion, having been chemically separated from the internal organism in the form of a gas, a liquid, or a solid ; it is the whole of the morbid action which is pressed from within—outward—and it is characteristic of a thorough and really curative treatment. The morbid action of the internal organism may continue either entirely or more or less in spite of this cutaneous eruption. Nevertheless, this eruption is always a favorable symptom ; it alleviates the sufferings of the patient, and generally prevents a more dangerous affection.

" The thorough cure of a widely ramified chronic disease in the organism is indicated by the most important organs being first relieved. The affection passes off in the order in which the organs have been affected, the more important being first relieved, the less important next, and the skin last.

" Even the superficial observer will not fail to recognize this law of order. An improvement, which takes place in a different order, can never be relied upon. A fit of hysteria may terminate in a flow of urine; other fits may terminate in the same way or in hemorrhage. The next succeeding fit shows how little the affection has been cured. The disease may take a different turn, it may change its form, and in this new form it may be less troublesome, but the general state of the organism will suffer in consequence of this transformation.

"Hence it is that Hahnemann inculcates with so much care the important rule to attend to the moral symptoms, and to judge of the degree of homœopathic adaptation existing between the remedy and the disease by the improvement which takes place in the moral condition and the general well-being of the patient.

"The law of order pointed out above accounts for the numerous cutaneous eruptions consequent upon homœopathic treatment, even where they have never been seen before; it accounts for the obstinacy with which many kinds of herpes and ulcers remain upon the skin, whereas others are dissipated like snow. Those which remain do so because the internal disease is yet existing. This law of order also accounts for the insufficiency of violent sweats, when the internal disease is not yet disposed to leave its hiding-place. It lastly accounts for one cutaneous affection being substituted for another.

"This transformation of the internal affection of such parts of the organism, as are essential to important functions, to a cutaneous affection (a transformation entirely different from the violent change effected by ointments, mustards, salves, etc.), is chiefly effected by anti-psoric remedies."

I may be excused for making this long quotation, perhaps already familiar to most of my readers, but these words of Hering seemed to me to convey to us all such sound advice that one could scarcely dwell too much or too long upon them, for to know the order of progress of curative action so as not to interfere with it is of vital importance. There is also another class of chronic disease, or at least may be so termed, which we often find in our practice; these are diseases caused by the suppression or bad treatment of acute disease. They are perhaps not so frequent now as formerly, but are still sufficiently numerous to merit our careful consideration. Hahnemann alludes to them, in paragraph 74, as follows: "Under the class of chronic disease, we have unfortunately to reckon those numerous factitious maladies, of universal propagation, arising from long-continued administration, by allopathists, of violent heroic medicines in large and increasing doses (from the abuse of Calomel, Corrosive sublimate, Mercurial ointment, Nitrate of Silver, Iodine and its ointment, Opium, Valerian, Quinine, Digitalis, Sulphur, etc., etc.), by which the vital power is either unmercifully weakened, or, if it be not indeed exhausted, becomes gradually so abnormally altered (in different manner, according to the particular medicine administered), that, in order to support life against such hostile and destructive assaults, it must effect changes in the organism, and either deprive this or that part of its sensibility or irritability, or exalt these properties to excess, produce dilatation or contraction, relaxation or induration of parts, or else totally destroy them, and here and there induce organic changes, both internally and externally (maim, as it were, the interior and exterior of the body), in order to protect the organization against entire destruction of life, from the reiterated assaults of such hostile and destructive influences." * * * "I regret to say that, when they have attained a considerable height, it would seem as if no remedy could be discovered or devised for their cure."

Such are the declarations of Samuel Hahnemann, and it will not do to pass them by as silly or absurd because the dominant school of medicine does not accept them as true. These views of the nature of chronic diseases have enabled physicians to make some of the grandest cures which have illumined the pages of medical history; and a careful study of these theories will enable us "to go and do likewise." One will easily see in them the causes of many of the chronic maladies which we have to combat—nervous diseases of all kinds, paralyses, tumors, uterine and ovarian ailments, thoracic disorders, etc., etc. In our attempts to treat these terrible diseases, we will meet with little success unless we are able to trace them back to their original cause. The ailment of to-day may have had its origin in, say, the abuse of Mercury years ago; or the chronic headaches you are now called upon to cure may be due to the scientific efforts, years ago, of some blundering gynæcologist. Imagine the condition of a psoric or syphilitic person whose constitution has been for years shattered by the gross treatment of a stupid allopathist; is it at all difficult to conceive that such causes may readily produce any chronic disease? E. J. L.

